# Hypofractionated radiosurgery for intact or resected brain metastases: defining the optimal dose and fractionation

**DOI:** 10.1186/1748-717X-8-135

**Published:** 2013-06-07

**Authors:** Bree R Eaton, Brian Gebhardt, Roshan Prabhu, Hui-Kuo Shu, Walter J Curran, Ian Crocker

**Affiliations:** 1 Departments of Radiation Oncology and the Winship Cancer Institute, Emory University, 1365 Clifton Rd. NE, Building A, Suite CT 104, Atlanta, GA, 30322, USA; 2 Current Affiliation: Medical College of Georgia, Augusta, GA, USA

**Keywords:** Hypofractionated, Radiosurgery, Brain, Metastases

## Abstract

**Background:**

Hypofractionated Radiosurgery (HR) is a therapeutic option for delivering partial brain radiotherapy (RT) to large brain metastases or resection cavities otherwise not amenable to single fraction radiosurgery (SRS). The use, safety and efficacy of HR for brain metastases is not well characterized and the optimal RT dose-fractionation schedule is undefined.

**Methods:**

Forty-two patients treated with HR in 3-5 fractions for 20 (48%) intact and 22 (52%) resected brain metastases with a median maximum dimension of 3.9 cm (0.8-6.4 cm) between May 2008 and August 2011 were reviewed. Twenty-two patients (52%) had received prior radiation therapy. Local (LC), intracranial progression free survival (PFS) and overall survival (OS) are reported and analyzed for relationship to multiple RT variables through Cox-regression analysis.

**Results:**

The most common dose-fractionation schedules were 21 Gy in 3 fractions (67%), 24 Gy in 4 fractions (14%) and 30 Gy in 5 fractions (12%). After a median follow-up time of 15 months (range 2-41), local failure occurred in 13 patients (29%) and was a first site of failure in 6 patients (14%). Kaplan-Meier estimates of 1 year LC, intracranial PFS, and OS are: 61% (95% CI 0.53 – 0.70), 55% (95% CI 0.47 – 0.63), and 73% (95% CI 0.65 – 0.79), respectively. Local tumor control was negatively associated with PTV volume (p = 0.007) and was a significant predictor of OS (HR 0.57, 95% CI 0.33 - 0.98, p = 0.04). Symptomatic radiation necrosis occurred in 3 patients (7%).

**Conclusions:**

HR is well tolerated in both new and recurrent, previously irradiated intact or resected brain metastases. Local control is negatively associated with PTV volume and a significant predictor of overall survival, suggesting a need for dose escalation when using HR for large intracranial lesions.

## Introduction

Brain metastases represent a significant cause of morbidity and mortality among cancer patients. Partial brain radiotherapy (RT) through stereotactic radiosurgery (SRS) is an important therapeutic tool for the treatment of brain metastases in multiple clinical settings. The addition of SRS to whole brain radiation (WBRT) may improve median survival and intracranial tumor control when compared to WBRT alone in select patients [1,2], is supported as an alternative to neurosurgical resection followed by WBRT [3,4], and is commonly used as salvage therapy for patients with intracranial tumor progression after WBRT. Additionally, SRS is increasingly used as a primary treatment modality in attempts to prevent or delay the known neurocognitive toxicities of WBRT [5]. Indeed, the benefits of radiosurgery are well recognized, but all patients are not considered acceptable candidates for SRS.

Brain metastases larger than 3 cm in diameter or producing more than 1 cm of midline shift are typically not considered acceptable candidates for single fraction radiosurgery [4] due to an insufficient volume response and an increased risk for cerebral edema following SRS [6-8]. Additionally, SRS may carry an increased toxicity risk when treating brain metastases of any size that are in close proximity to critical structures, such as the optic chiasm or brainstem, and when the location of the metastasis has previously received high radiation doses. In these instances, hypofractionated radiosurgery (HR) delivered in 3-5 fractions over multiple days is an alternative therapeutic option for delivering partial brain RT. HR may provide the benefit of improved local control and reduced neurocognitive decline when compared to WBRT, with reduced toxicity risk when compared to SRS for large intracranial lesions or metastases in sensitive locations. The use, safety and efficacy of HR is supported by previously published series with short-term follow up and varying radiation dose and fractionation schedules described [9-19]. However, data reporting long-term outcomes is limited and the optimal dose-fractionation schedule for HR is undefined. The purpose of this analysis is to evaluate the relationship between RT dose-fractionation and clinical outcomes in a cohort of patients with long-term imaging follow-up, who were treated with HR at Emory University for brain metastases not amenable to SRS.

## Materials and methods

### Patient selection

With institutional review board approval, the treatment records from the Radiation Oncology Department of the Winship Cancer Institute of Emory University were searched for patients having completed a planned course of hypofractionated radiation therapy to the brain consisting of >1 and ≤ 5 fractions. Patients with a diagnosis of secondary malignant neoplasms of the brain, who were treated with or without surgical resection, were included whether they had or had not received prior radiation therapy to the treatment site. All treated patients had a prior pathologic diagnosis of malignancy. Patients without follow-up diagnostic MRI for outcome assessment were excluded from analysis.

### Hypofractionated radiation therapy

Patients underwent high-resolution treatment planning MRI scan with and without contrast immediately before or following CT simulation. Simulation was performed in the supine position using an Aquaplast mask (WFR/Aquaplast Corp., Wyckoff, NJ) for immobilization. The treatment planning MRI was rigidly registered to the simulation CT for improved target and normal tissue delineation. For patients with intact brain metastases, the gross tumor volume (GTV) was defined as the contrast enhancing abnormality on the T1 post-contrast MRI sequence. For patients who had prior resection, the GTV was designed to include the resection cavity as well as any enhancing tumor. The clinical tumor volume (CTV) was defined by expansion of the corresponding GTV with 0 - 7 mm margin to account for potential microscopic tumor extension and was modified according to how well the tumor was visualized and the anatomic location of the tumor to respect natural barriers to tumor spread such as bone. A planning target volume (PTV) was created to account for patient setup uncertainty and target motion by the addition of a 0 – 3 mm margin to the CTV. Median total margins for GTV to PTV expansion was 2 mm (range 0 - 10 mm). Zero expansion margin was used in 2 patients who had previously received radiation therapy and in which the GTV abutted the brainstem. Conversely, the largest total expansion margin of 10 mm (7 mm GTV to CTV and 3 mm CTV to PTV) was used in one patient with a poorly visualized resection cavity.

Radiation therapy dose and fractionation was prescribed at the discretion of the treating physician. For the purpose of dose-response analysis, radiation therapy dose was converted to biological equivalent dose (BED) [20] and single fraction equivalent dose (SFED) [21] using the following equations where D equals total dose and d equals dose per fraction, assuming an α/β ratio of 10 for tumor control and a Dq of 1.8 [21].

$$\mathrm{BED}=D*\left[1+d/\left(a/\beta \right)\right]$$

$$\mathrm{SFED}=D-\left(n-1\right)*\mathrm{Dq}$$

The prescription dose was prescribed such that at least 95% of the PTV received 100% of the prescribed dose. Registration and contouring were carried out with Velocity AI (Velocity Medical Systems, Atlanta, GA) and dose calculation was done with Eclipse (Varian Medical Systems, Palo Alto, CA). Patients underwent cone beam CT scans using 6 degrees of freedom registration for precise positioning prior to therapy [22]. Multifield (>10 fields) intensity modulated radiotherapy (IMRT) or dynamic conformal arcs (DCA) with photons of 6MV were used for treatment delivery.

### Follow-up and outcomes analysis

Patients were routinely followed by history, physical exam and MRI, with and without contrast, initially at 4-6 weeks after completion of radiotherapy and every 2-3 months thereafter, or at the first incidence of symptomatic progression. Intracranial failure was defined as tumor recurrence or progression within the brain, as was evident by diagnostic MRI. Failure was considered local if >90% of the tumor recurrence was within the prescription isodose volume, distant if >90% of the recurrent tumor was outside the prescription isodose and marginal if neither local nor distant. Radiation necrosis was diagnosed by increased enhancement on T1 post-contrast MRI with or without surrounding abnormal T2/FLAIR signal abnormality, which corresponded with hypoperfusion evident on dynamic susceptibility contrast perfusion images and did not progress rapidly on serial MRIs, or was confirmed by surgical specimen. Patients with tumor progression and/or radiation necrosis were frequently reviewed at central nervous system multi-disciplinary tumor board for consensus diagnosis.

### Statistical analysis

Descriptive statistics were compiled to characterize the patient population. Kaplan-Meier analysis was used to estimate overall survival and freedom from local failure, intracranial progression, and radiation necrosis. Survival analysis was calculated from time of radiation therapy initiation until first evidence of local failure, intracranial failure, or death. For alive patients, overall survival was censored at last follow-up, and intracranial progression free survival, local control, and freedom from radiation necrosis were censored at last follow-up MRI of the brain or at the time of subsequent WBRT. Cox-regression analysis was used to assess the significance of multiple radiation therapy and tumor variables, including GTV/cavity size, PTV volume, dose per fraction, total dose, BED_10_, SFED, previous radiation therapy received, and resected vs. unresected tumor on local control, overall survival, and freedom from radiation necrosis. All statistical tests were 2 sided and performed using SPSS software (IBM SPSS version 19.0, Chicago, IL).

## Results

### Patient population

Forty-seven patients treated with HR for new or progressive brain metastases or resection cavities between May 2008 and August 2011 at the Emory Clinic were identified, and 42 of these patients were included for outcomes analysis. Five patients were excluded due to lack of post-treatment imaging follow-up. Table 1 includes patient, tumor and previous treatment characteristics. The most common indication for the use of HR was large tumor or resection cavity size. Additionally, patients not considered acceptable candidates for single fraction SRS due to close proximity of the tumor to critical structures such as the optic chiasm, optic nerves, or brainstem were also included.

**Table 1:** Patient, tumor and previous RT characteristics

**n = 42 patients**	**n (%)Median (range)**
**Age**	58 years (23-81)
**Gender**	
Male	19 (45%)
Female	23 (55%)
**Performance Status**	
ECOG 0-2	37 (88%)
ECOG 3-4	5 (12%)
**Primary Cancer Diagnosis**	
Breast	12 (29%)
Lung, NSCLC	10 (24%)
Melanoma	9 (21%)
Head and Neck	5 (12%)
Other/Unknown	6 (14%)
**Intact Brain Metastasis**	20 (48%)
**Resection Cavity**	22 (52%)
GTR	11
STR	11
**Target Volume**	
GTV/Cavity Maximum Dimension	3.9 cm (0.8-6.4 cm)
GTV/Cavity Volume	13.6 cc (0.2 – 57.0 cc)
PTV Volume	24.5 cc (0.8 – 122.0 cc)
**Previous RT and Dose to HR site**	
None	20 (48%)
WBRT	5 (12%)
	37.5 Gy (30-45)
SRS	5 (12%)
	18 Gy (5-21)
IMRT	4 (10%)
	47.5 Gy (10-60)
Combination	8 (19%)
	41.25 Gy (10 -75)
**Time from Previous RT to HR**	13 months (0.3-32.4)

*Abbreviation:* NSCLC, non-small cell lung cancer.

### Radiation therapy

Total radiation dose delivered ranged from 21-30 Gy, in 5-7 Gy per fraction for 3-5 fractions over a median treatment time of 7 days (range 3 – 14). In 4 patients status-post a subtotal resection, a simultaneous infield boost of an additional 1 – 1.5 Gy per fraction was delivered to the gross residual disease. Table 2 lists the dose-fractionation schedules used. All patients completed the prescribed radiation course as planned.

**Table 2:** HR fractionation schedules

**Fractionation schedulen = 42 patients**	**n (%)**	**BED**_**10**_	**SFED(Gy)**
7 Gy x 3*	28 (67)	37.5	17.4
6 Gy x 4**	6 (14)	38.4	18.6
6 Gy x 5	5 (12)	48	22.8
5 Gy x 5	2 (4)	37.5	17.8
5 Gy x 6	1 (2)	45	21

*Three patients status-post subtotal resection *(STR)* received 7 *Gy x 3* to the *PTV* with a simultaneous infield boost *(SIB)* to the *GTV* for an additional 1 *Gy* per fraction. ** One patient status-post *STR* received 6 *Gy x* 4 to the *PTV* with a *SIB* to the *GTV* for an additional 1.5 Gy per fraction.

### Intracranial control and patterns of failure

**Figure 1:**
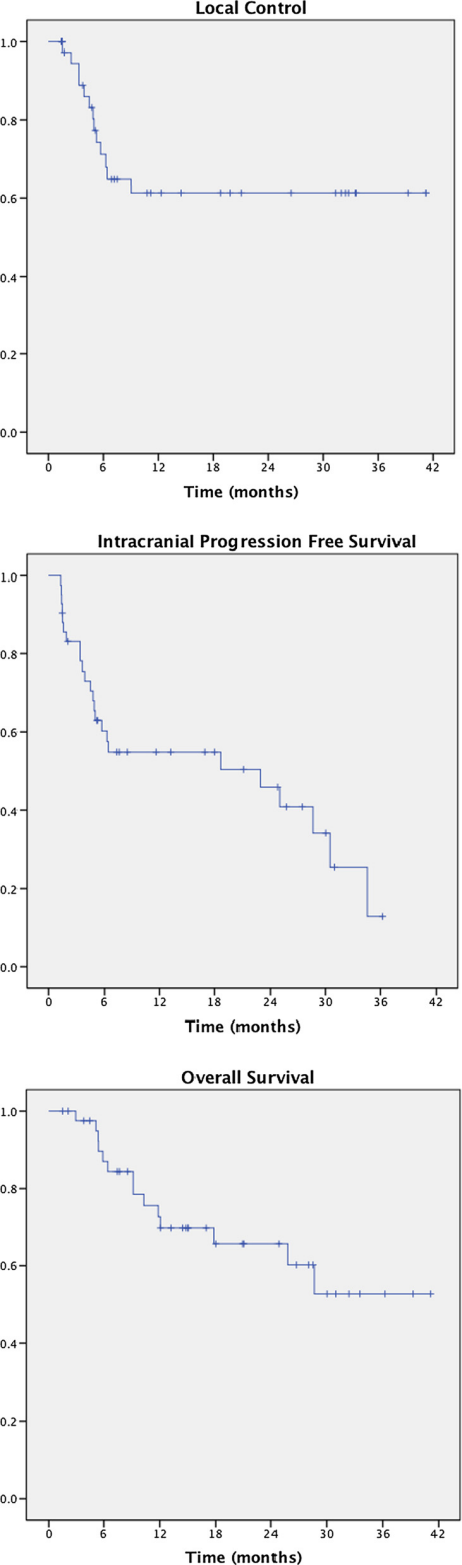
Kaplan-Meier curves of local control, freedom from intracranial progression and overall survival.

The median imaging follow-up of this study was 8 months (range 1- 41). KM estimates of 1 year LC and intracranial PFS are 61% (95% CI 0.53 – 0.70) and 55% (95% CI 0.47 – 0.63), respectively (Figure 1). On univariate analysis, local failure was significantly associated with GTV/Cavity maximum dimension (p = 0.037) and PTV volume (p = 0.007, Table 3). No statistically significant association was found between LC and dose per fraction, total dose, biological equivalent dose (BED), single fraction equivalent dose (SFED), previous RT received, or resected vs. unresected tumor (Table 3). Multivariate analysis was not performed given the only significant variables were both measures of tumor size.

**Table 3:** Cox-regression analysis of the relationship between freedom from local failure and multiple tumor and RT variables

**Variable**	**Freedom from local failure**
	**Uni-variate**
	**(p)**
**GTV/Cavity Max Dimension**	**0.037**
**PTV Volume**	**0.007**
**Dose per fraction**	0.106
**Total Dose**	0.761
**SFED**	0.321
**BED**_**10**_	0.887
**First RT vs. Salvage RT**	0.241
**Cavity vs. Solid Tumor**	0.992

At last follow-up, 18 patients (48%) were free from any intracranial failure. Among the 24 patients with intracranial progression, site of first recurrences was isolated distant failure in 13 (50%), local failure in 5 (23%), marginal failure in 1 (2%), and distant failure occurring simultaneously with local failure in 4 (18%) or with marginal failure in 1 (2%). Distant intracranial failure was thus the predominant site of first intracranial progression, occurring in 16 patients (73%).

In total, 10 patients received salvage radiotherapy for intracranial tumor progression after HR. Three patients (7%), two with local and distant intracranial failure and one with distant intracranial failure alone, received salvage WBRT at a median time of 4.7 months (range 3-7) after completion of HR. Six patients (14%), one with local and distant intracranial failure and 5 with distant intracranial failure alone, receive salvage SRS at a median time of 15.5 months (range 2- 34) after completion of HR. One patient with local and distant intracranial failure received salvage WBRT with SRS boost to the site of distant failure at 1.5 months after completion of HR. Seven patients with local intracranial failure did not receive salvage RT to the local site due to the high cumulative dose received between HR and previous brain RT (median 76 Gy, range 60 - 85.5 Gy), and two patients with local intracranial failure did not receive salvage local RT due to systemic disease progression and poor prognosis.

### Overall survival

**Figure 2:**
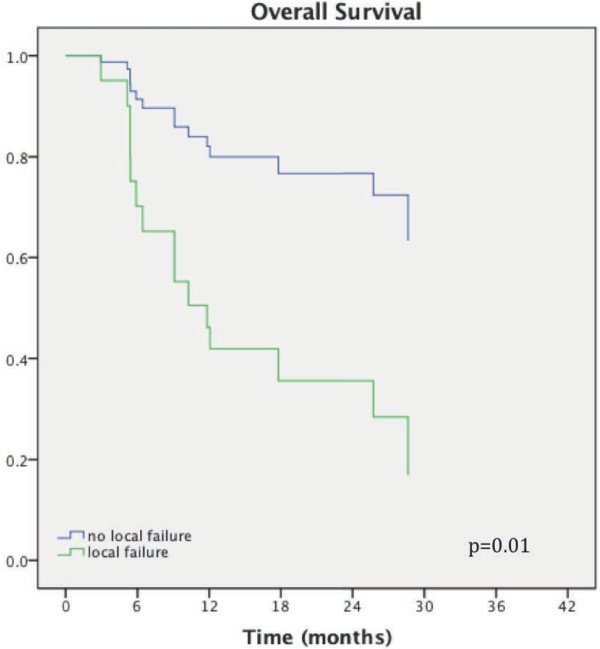
Kaplan-Meier curve of overall survival in relationship to local tumor control.

After a median follow-up of 15 months (range 2 - 41 months), median survival has not been reached. KM estimate 1 year overall survival is 73% (95% CI 0.65 – 0.79, Figure 2). Survival was significantly associated with whether HR was delivered as first therapy or salvage treatment after prior RT (p = 0.04) and with local tumor control (p = 0.01) on univariate analysis. After accounting for initial versus salvage treatment, local control remained a significant predictor of OS (HR 0.57, 95% CI 0.33 - 0.98, p = 0.04). Patients with local failure had a median OS of 6 months, while median survival was not reached in patients without local failure. At last follow-up, 27 (64%) are alive and 15 of the alive patients (56%) have stable intracranial disease. Fifteen patients (36%) have died, of whom 9 patients (60%) have died with progressive intracranial disease.

### Treatment toxicity

**Figure 3:**
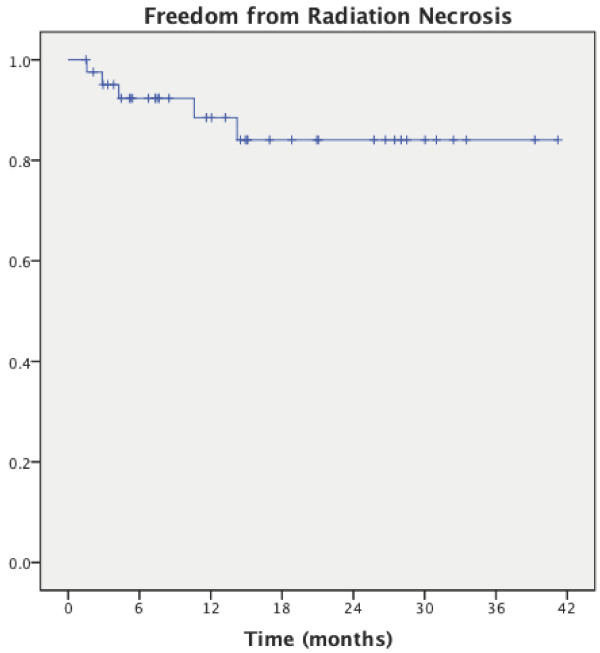
Kaplan-Meier curve of freedom from radiation necrosis.

The KM estimate of freedom from radiation necrosis at 1 year is 90% (CI 0.84 – 0.96), (Figure 3). No association was found between the incidence of radiation necrosis and previous RT received, total dose, dose per fraction, BED, SFED, PTV volume, or resection cavity vs. solid tumor. Four patients (9.5%) were diagnosed with radiation necrosis by follow-up imaging, and 3 of these patients (7%) were symptomatic requiring oral steroids. One asymptomatic patient underwent surgical resection for suspected tumor recurrence and was found to have radiation necrosis on final pathology.

## Discussion

With long-term follow-up, our data demonstrates that HR is well tolerated in both new and previously irradiated, intact or resected brain metastases and represents a promising therapeutic option for these patients who are not otherwise considered candidates for single fraction SRS. One-year estimates of local control, intracranial progression free survival and overall survival in our series were 61%, 55%, and 73%, respectively, while the rate of symptomatic radiation necrosis was only 7% (3 of 42 patients). Thus, our data adds to the previously published literature supporting the use of HR for brain metastases and represents excellent outcomes considering the fact that the tumors in our series were considerably larger than in other reported series and were heavily pre-treated, with the majority having received prior radiation therapy to the HR treatment site (Table 4).

**Table 4:** Published HRr series with local control and toxicity data

**Series**	**N (patients/metastases)**	**Lesion size(median)**	**Fractionation**	**Local control**	**Reported toxicity**
**Aoki et al. [**[7]**]**	44/65	-	5-6 Gy x 3-5	72% at 1 year	2% grade 3
**Aoyama et al. [**[8]**]**	87/159	3.3 cc	8.75 Gy x 4	81% at 1 year	7% grade 3
**DePotter et al. [**[9]**]**	35/58	8.6 cc	WBRT + 6 Gy x 5	66% at 1 year	11% grade 3
**Fahrig et al. [**[17]**]**	150/228	6.1 cc	6-7 Gy x 5	-	22%
			5 Gy x 7	-	7%
			10 Gy x 4	-	0%
**Giubilei et al. [**[10]**]**	30/44	2.1 cm/4.8 cc	6 Gy x 3/ 8 Gy x 4	86% at 1 year	-
**Kwon et al. [**[11]**]**	27/52	1.2 cm/0.5 cc	20-35 Gy in 4-6	68% at 1 year	5.8% radiation necrosis
**Lindvall et al. [**[12]**]**	47/47	-	8 Gy x 5	84% crude	6.25% radiation necrosis, 1 patient death
**Narayana et al. [**[13]**]**	20/20	-	6 Gy x 5	70% at 1 year	15%
**Ogura et al. [**[14]**]**	39/46	1.8 cm	7 Gy x 5 or	17% at 1 year	2.5% grade 3
			WBRT + 4-5 Gyx5		
**Wang et al. [**[15]**]**	37/37	cavity > 3 cm	8 Gy x 3	80% at 6 months	9%
**Current Study**	42/42	3.9 cm/13.6 cc	5-8 Gy x 3-5	62% at 1 year	7% radiation necrosis

We found local control to be negatively associated with PTV volume. This finding is in agreement with previously published analyses of both SRS and HR, which demonstrated decreased local control and tumor response with larger PTV volumes [7,13]. In this series, the median GTV to PTV expansion was only 2 mm. Thus the PTV volume was predominantly reflective of GTV size, which was also found to be negatively associated with local control. In total, in-field tumor recurrence occurred in 9 patients with only 2 patients experiencing tumor recurrence marginal to the treatment volume. The predominance of in-field local recurrences suggests marginal misses due to insufficient expansion margins is not a major contributor to the incidence of tumor recurrence. Indeed, the frameless radiosurgery technique with 6 degrees of freedom registration used herein has previously been demonstrated to have an accuracy and reproducibility of less than 1 mm [22,23]. The importance of precise positioning and immobilization in the treatment of these large intracranial lesions with highly conformal RT is important for both maintaining tumor control and minimizing the dose delivered to normal brain.

Although higher dose per fraction was not associated with improved local control, this may be explained by the fact that the higher dose per-fraction regimen most commonly used in this series, 7 Gy x 3, has a lower BED and SFED compared with the other regimens used (Table 2). In the only other published HR series to compare different dose-fractionation schemes, Fahrig et al. [19] demonstrated higher rates of complete response in tumors treated with 6-7 Gy x 5 or 5 Gy x 7 versus 4 Gy x 10. These findings suggest dose escalation may be of benefit when using HR for larger intracranial tumors. The significant association between local tumor control and overall survival in this series further highlights the need for dose optimization.

In the only prospective trial of HR for the treatment of brain metastasis, Ernst-Stecken et al. [18] used either HR alone (7 Gy x 5) or WBRT followed by an HR boost (boost dose 6 Gy x 5) for patients with 1-4 brain metastases larger than 3 cc and controlled extracranial disease. Results revealed a 2.8% rate of progressive disease after a median follow-up of 7 months. This rate of local failure is considerably lower than has been demonstrated in our and other retrospective series, and appeared to be associated with increased toxicity as 49% of patients (25 of 51) experienced symptoms associated with radiologic signs of increased edema requiring steroid medication. From this analysis, it was found that the volume of normal brain receiving ≥ 4 Gy per fraction (V_4Gy_) was significantly associated with the incidence of radiation necrosis. In pursuit of the excellent tumor control demonstrated in this Phase II trial, a prospective phase I dose-escalation trial performed within the limitations of the aforementioned dose constraint is required to determine the maximum tolerated dose, maximum tolerated dose that will maximize patient benefits and minimize associated risks in the use of HR for large brain metastases. With this purpose in mind, we have initiated an institutional phase-1 dose escalation protocol of HR for large brain metastasis ≥ 3 cm and ≤ 6 cm to be treated in 5 fractions, delivered in 2-3 fractions per week (NCT01705548).

Our data is limited in that the population is small and heterogeneous and the analysis is subject to the multiple potential confounding biases and limitations inherent to any retrospective review. Safety may be overestimated in a retrospective study and toxicity analysis is primarily limited to clearly documented diagnoses of radiation necrosis. However, the long-term follow up with post-treatment MRI imaging is a strength of this analysis. The various dose-fractionation schedules used in our series gave us the unique ability to compare clinical outcomes with RT dose, and although no significant difference in local control or radiation necrosis were seen among the patients treated with the different dose-fractionation regimens, in light of the higher BED and SFED of 6 Gy x 5 and the results demonstrated with this regimen in our series and others [7,9,17], we recommend 6 Gy x 5 as the future HR treatment schedule used outside of a prospective protocol.

## Conclusions

The use of hypofractionated radiosurgery for the treatment of both new and previously irradiated, intact or resected, brain metastases is well tolerated and leads to good local tumor control with excellent overall survival. Local control was negatively associated with PTV volume and significantly predicted for overall survival. We recommend 6 Gy x 5 be used to deliver HR for large intracranial tumors and further dose escalation in a controlled, prospective trial may be of benefit.

## Competing interest

Dr. Crocker is a Co-Founder and Shareholder of Velocity Medical Solutions. He is entitled to royalties on sales based on an Intellectual Property Agreement between Emory University and Velocity Medical Solutions.

## Authors’ contributions

BE, RP, and IC contributed to the conception and design of the study. BE, BG, RP, and IC acquired, analyzed and interpreted the data. BE, BG, RP, HS, WC, and IC were involved in the critical appraisal, drafting, and revising of the manuscript. All authors approved the final manuscript.
